# Effects of Protease-Hydrolyzed and *Cordyceps militaris*-Fermented Sea Cucumber Viscera Hydrolysates on Cellular Function and Energy Metabolism in HCT116 Cells

**DOI:** 10.3390/foods15173065

**Published:** 2026-08-29

**Authors:** Biyi Chen, Yuyang Li, Xinghe Chen, Xinyue Jia, Ying Li, Rui Mi, Zunchun Zhou

**Affiliations:** 1 Key Laboratory of Germplasm Improvement and Fine Seed Breeding for Marine Aquatic Animals, Liaoning Ocean and Fisheries Science Research Institute, Dalian 116023, China; cbysdsu2020@163.com (B.C.);; 2 College of Food Science and Engineering, Dalian Ocean University, Dalian 116023, China

**Keywords:** sea cucumber viscera hydrolysates, *Cordyceps militaris* fermentation, HCT116 cells, cellular function, energy metabolism

## Abstract

Colorectal cancer (CRC) remains a major global health concern. Marine by-products are increasingly recognized as promising sources of functional ingredients with diverse bio-activities. This study investigated the effects of sea cucumber viscera hydrolysates prepared by protease hydrolysis (SVH) and further fermented with *Cordyceps militaris* (FSVH) on cellular function and energy metabolism in HCT116 cells. Cell viability, apoptosis, migration, and invasion were evaluated using CCK-8, flow cytometry, wound healing, and Transwell assays. Cellular energy metabolism was assessed by measuring glucose uptake, lactate production, oxygen consumption rate (OCR), and extracellular acidification rate (ECAR). The results showed that SVH and FSVH exhibited distinct biological activity profiles in HCT116 cells. Both SVH and FSVH induced apoptosis and inhibited the migration and invasion of HCT116 cells. Both treatments affected glucose metabolism and mitochondrial respiratory function, including glucose uptake, lactate production, mitochondrial respiration, and glycolytic activity. SVH exhibited stronger inhibitory effects on glycolysis-related parameters, whereas FSVH showed greater effects on mitochondrial respiratory capacity. These findings indicate that *C. militaris* fermentation modified rather than simply enhanced the biological properties of sea cucumber viscera hydrolysates, resulting in distinct effects on cellular function and energy metabolism. This study highlights the potential application of sea cucumber viscera hydrolysates for the value-added utilization of marine processing by-products.

## 1. Introduction

Colorectal cancer (CRC) is one of the most common and deadly malignancies worldwide [1,2]. Although current therapeutic strategies have improved CRC management, limitations such as systemic toxicity and drug resistance remain major challenges [1,3]. Therefore, natural products with high bioactivity and relatively low toxicity have attracted increasing attention as sources of dietary supplements and complementary therapeutic agents for CRC [4,5,6], including bioactive compounds from microorganisms (e.g., fungi) and marine organisms [7,8,9].

Sea cucumbers (e.g., *Apostichopus japonicus*) are traditional marine foods with both nutritional and medicinal values and are widely consumed in Asian countries. The body wall is the primary edible portion and has been extensively studied for its health benefits [10,11]. China has developed a large-scale sea cucumber aquaculture industry, with cultured sea cucumber production reaching 326,172 t in 2024, and Liaoning Province contributing approximately 35.7% of the national output [12]. However, the rapid expansion of sea cucumber production and processing has generated substantial visceral by-products. Sea cucumber viscera, including intestines, gonads, respiratory trees, Cuvierian tubules, and other internal structures, may account for up to approximately 50% of the total biomass [13]. These by-products are often discarded, resulting in resource waste and environmental burden.

Sea cucumber viscera are rich in proteins, polysaccharides, saponins, fatty acids, and other bioactive compounds, with compositions comparable to those of the body wall [13,14,15]. Comparative analysis of enzymatic hydrolysates from sea cucumber body wall and viscera showed that proteins/peptides were the predominant components in both tissues, accounting for 50.62% and 49.12%, respectively, while viscera hydrolysates also contained considerable levels of total sugars (11.67%), crude lipids (9.64%), and ash (10.28%) [16]. These components may contribute to the reported antioxidant, immunomodulatory, anti-fatigue, and antitumor activities [17,18,19,20]. However, some bioactive compounds exist in macromolecular forms with limited bioavailability, restricting their practical application. Enzymatic hydrolysis has therefore been widely used to release bioactive peptides and improve the utilization of protein-rich marine by-products [21]. Accordingly, sea cucumber viscera hydrolysates provide a promising substrate for further functional modification and the development of marine-derived bioactive products.

*Cordyceps militaris* is an edible and medicinal fungus traditionally used in Asian countries and recognized for its pharmacological potential due to its diverse bioactive constituents, including cordycepin, polysaccharides, and bioactive peptides derived from its proteins [22]. These compounds have been reported to exhibit antioxidant, immunomodulatory, and antitumor activities [22,23]. For example, the *C. militaris*-derived biomimetic anticancer peptide C-ori, identified through in silico screening of the *C. militaris* pepsinized peptidome, significantly inhibited the growth of human colorectal adenocarcinoma HT-29 cells by inducing apoptosis [24]. In addition, cordycepin produced during *C. militaris* fermentation has demonstrated anticancer effects in various cancer models [25], including inhibition of colon cancer cell proliferation, migration, and invasion [26].

Microbial fermentation has been increasingly applied for the value-added utilization of food-processing by-products because microbial metabolism can transform substrate components, promote the release or production of bioactive compounds, reduce undesirable substances, and improve biological and sensory properties [23,27]. Therefore, fermentation with *C. militaris* may not only modify the functional properties of sea cucumber viscera hydrolysates through microbial transformation but also introduce fungal-derived bioactive metabolites, such as cordycepin, while potentially improving other quality-related characteristics. Previous studies have shown that fermentation of sea cucumber intestines generated antioxidant peptides with cytoprotective effects [20]. Our previous study also found that *C. militaris*-fermented sea cucumber viscera hydrolysates exhibited antioxidant activity and alleviated oxidative damage in an oxidative stress-induced Caco-2 cell model [28]. Together, enzymatic hydrolysis combined with *C. militaris* fermentation may represent an effective strategy to modify the biological properties and enhance the utilization value of sea cucumber by-products.

The human colorectal cancer HCT116 cell line is widely used as an in vitro model for investigating CRC-associated cellular behaviors and metabolic characteristics [29]. CRC progression is characterized by dysregulated cell proliferation, apoptosis resistance, enhanced migration and invasion, and metabolic reprogramming [30]. To sustain rapid growth and survival, cancer cells typically undergo alterations in glucose metabolism and mitochondrial function [31]. Therefore, comparing the effects of fermented and non-fermented sea cucumber viscera hydrolysates on cellular functions and energy metabolism in HCT116 cells may provide valuable insights into how different processing strategies influence the biological properties of these hydrolysates.

Although the bioactivities of sea cucumber viscera and *C. militaris* have been investigated separately, limited information is available on how *C. militaris* fermentation alters the biological properties of protease-hydrolyzed sea cucumber viscera and their effects on colorectal cancer cell function and energy metabolism. In this study, fermented sea cucumber viscera hydrolysates (FSVH) were obtained by fermenting protease-hydrolyzed sea cucumber viscera hydrolysates (SVH) with *C. militaris*. The aim of this study was to comparatively evaluate the biological effects of SVH and FSVH on cellular functions and energy metabolism in HCT116 colorectal cancer cells. This study provides insights into the influence of *C. militaris* fermentation on the biological properties of sea cucumber viscera hydrolysates and their potential application as functional food ingredients.

## 2. Materials and Methods

**Figure 1:**
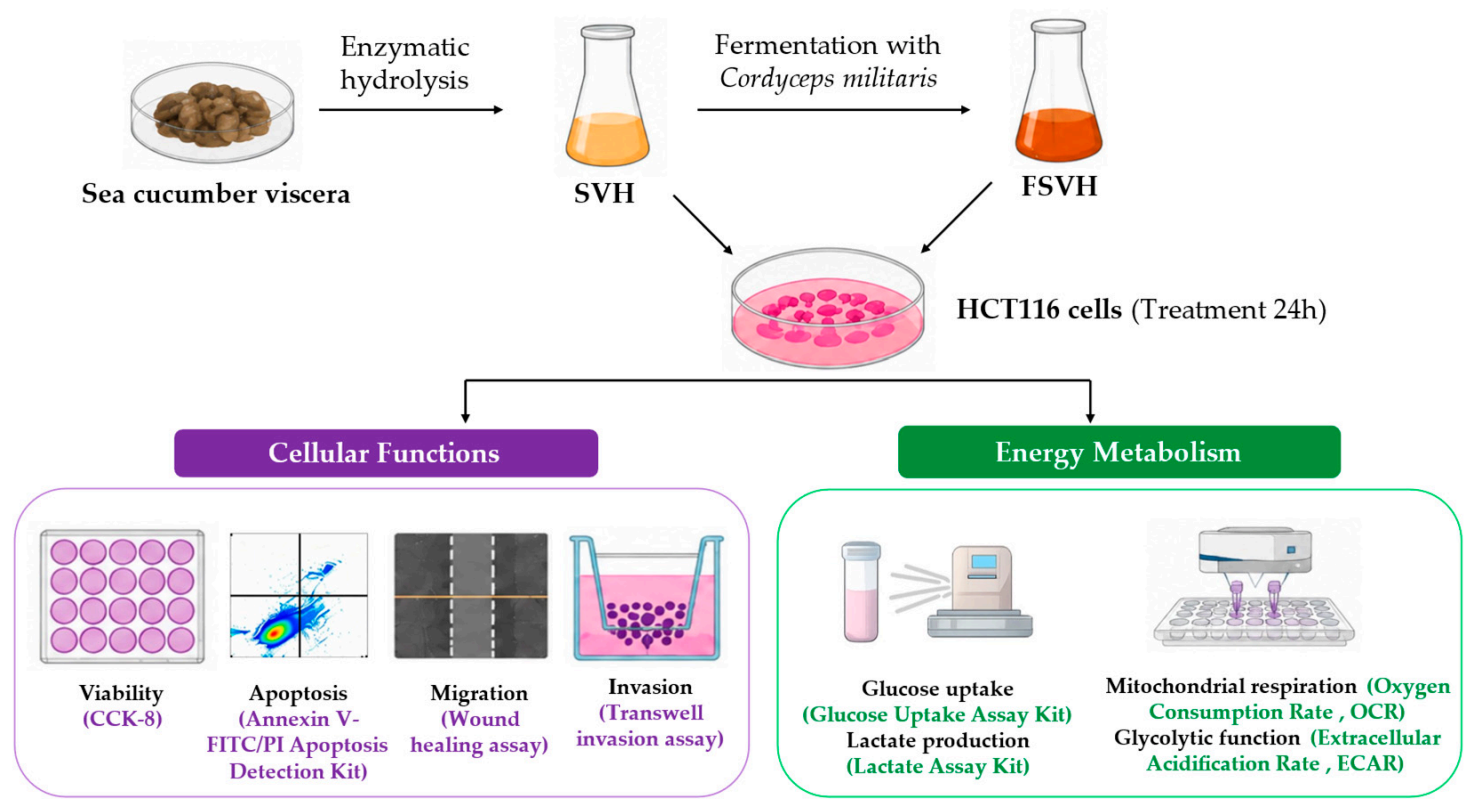
Graphical overview of the experimental workflow.

A graphical overview summarizing the experimental workflow is presented in Figure 1. This figure illustrates the sequential steps of SVH and FSVH preparation, HCT116 cell treatment with SVH and FSVH, and subsequent assays for cellular functions (viability, apoptosis, migration, and invasion) and energy metabolism (glucose uptake, lactate production, mitochondrial respiration, and glycolytic function).

### 2.1. Preparation of SVH and FSVH

SVH was prepared by enzymatic hydrolysis of sea cucumber viscera, and FSVH was obtained by fermenting SVH with *C. militaris* using SVH as the fermentation substrate. Briefly, sea cucumber viscera were soaked in distilled water for 8 h, and this process was repeated three times to remove residual salt. The desalted viscera were then homogenized with distilled water and hydrolyzed with papain (105 U/g substrate) at 50 °C for 2 h under magnetic stirring. The hydrolysate was subsequently filtered, clarified, and sterilized at 115 °C for 30 min to obtain SVH.

SVH was then inoculated with an activated *C. militaris* liquid culture and fermented at 22 °C for 7 days under aeration and agitation. After fermentation, the broth was sterilized at 115 °C for 30 min. The supernatant was collected by centrifugation and freeze-dried to obtain FSVH.

The compositional characteristics of SVH and FSVH were reported in our previous study [28]. Briefly, compared with SVH, FSVH showed a slightly lower protein/peptide content and a slightly higher saponin content, although neither difference was statistically significant, while the polysaccharide content was significantly higher (*p* < 0.05). Moreover, cordycepin was detected only in FSVH after *C. militaris* fermentation. The detailed compositional characteristics of SVH and FSVH are provided in Appendix A.

### 2.2. Cell Culture and Treatment

The human colorectal cancer HCT116 cell line was obtained from Procell Life Science (CL-0096, Wuhan, China). Cells were cultured in complete Dulbecco’s Modified Eagle Medium (DMEM; KGM12800S, KeyGEN BioTECH, Nanjing, China) and maintained at 37 °C in a humidified incubator containing 5% CO_2_ (BPN-80CW, Shanghai Yiheng Scientific Instruments Co., Ltd., Shanghai, China).

Cells were treated with SVH and FSVH at different concentrations (0, 0.25, 0.5, 1, 1.5, and 2 mg/mL), prepared based on the dry weight of the freeze-dried hydrolysate samples, for 24 h according to our previous study with modifications [28]. Based on preliminary screening of cell viability and apoptosis responses, concentrations that induced detectable apoptotic responses while maintaining relatively acceptable cell viability were selected for subsequent experiments. SVH at 1.5 mg/mL and FSVH at 1.0 mg/mL were selected as representative concentrations for further evaluation of cellular functions and energy metabolism. Untreated cells were used as the control group.

### 2.3. Cellular Function Assays

#### 2.3.1. Cell Viability Assay

Cell viability was assessed using the Cell Counting Kit-8 (CCK-8; KGA317-1, KeyGEN BioTECH, Nanjing, China). HCT116 cells were seeded into 96-well plates and treated with different concentrations of SVH and FSVH for 24 h. Subsequently, 10 μL of CCK-8 solution was added to each well and incubated at 37 °C for 2 h. Absorbance was measured at 450 nm using a microplate reader (WD-2012B, Beijing Liuyi Biotechnology, Beijing, China) [32].

#### 2.3.2. Apoptosis Assay

Cell apoptosis was measured using the Annexin V-FITC/PI Apoptosis Detection Kit (AP101-100-kit, Multi Sciences, Hangzhou, China). After treatment with SVH or FSVH for 24 h, cells were collected, washed with phosphate-buffered saline (PBS; KGB5001, KeyGEN BioTECH, Nanjing, China), and resuspended in 300 μL binding buffer. Cells were then stained with 5 μL Annexin V-FITC and 10 μL propidium iodide (PI) for 10 min at room temperature in the dark. The stained samples were analyzed using a flow cytometer (NovoCyte 2060R, Acea Biosciences, Hangzhou, China) [33].

#### 2.3.3. Wound Healing Assay

Cell migration ability was evaluated using a wound healing assay. HCT116 cells were cultured until over 90% confluence, and a linear scratch was generated using a sterile 10 μL pipette tip. After washing with PBS, cells were cultured in serum-free medium. Images were captured at 0, 24, and 48 h using an inverted microscope (MF53, Guangzhou Micro-shot Technology Co., Ltd., Guangzhou, China). The wound closure rate was quantified using ImageJ software (version 1.54, National Institutes of Health, Bethesda, MD, USA).

#### 2.3.4. Transwell Invasion Assay

Cell invasion ability was evaluated using a Transwell invasion assay [32]. Cells were suspended in serum-free medium and seeded into the upper chamber at a volume of 0.3 mL, while 0.5 mL of complete medium was added to the lower chamber. After incubation for 24 h, non-invading cells were removed, and invading cells were stained with 0.1% crystal violet (G1061, Solarbio, Beijing, China) for 1 h. Cells were observed and photographed using a microscope (BX43, Olympus, Tokyo, Japan). After staining was eluted with 33% acetic acid, absorbance was measured at 562 nm.

### 2.4. Energy Metabolism Assays

#### 2.4.1. Glucose Uptake and Lactate Production Assays

Glucose uptake was evaluated using a Glucose Uptake Assay Kit (ab136955, Abcam, Cambridge, UK) according to the manufacturer’s instructions. After treatment, cells were washed and incubated with assay reagents, and absorbance was continuously recorded at 412 nm every 2–3 min using a microplate reader. This assay is based on the uptake of 2-deoxyglucose (2-DG), which is phosphorylated intracellularly to 2-deoxyglucose-6-phosphate (2-DG6P). Intracellular 2-DG6P levels were quantified according to a 2-DG6P standard curve and used as an indicator of glucose uptake.

Lactate production was evaluated using a Lactate Assay Kit (A019-2-1, Nanjing Jiancheng Bioengineering Institute, Nanjing, China) according to the manufacturer’s instructions. After treatment, cell lysates were collected and incubated with assay reagents for color development. Absorbance was measured at 530 nm, and lactic acid concentration was calculated according to a lactic acid standard curve.

#### 2.4.2. Measurement of Oxygen Consumption Rate (OCR) and Extracellular Acidification Rate (ECAR)

The effects of SVH and FSVH on mitochondrial respiration and glycolytic function were evaluated by measuring OCR and ECAR using a Seahorse XFe24 Extracellular Flux Analyzer (Agilent Technologies, Santa Clara, CA, USA). XF Cell Mito Stress Test Kit (103015-100, Agilent Technologies, Santa Clara, CA, USA) and XF Glycolysis Stress Test Kit (103344-100, Agilent Technologies, Santa Clara, CA, USA) were used according to the manufacturer’s instructions. HCT116 cells were seeded at an equal density of 1 × 10^4^ cells/well before Seahorse analysis, and OCR and ECAR values were normalized to the initial cell number per well.

For OCR measurement, oligomycin was injected at 30 min to inhibit ATP synthase, followed by FCCP at 60 min to achieve maximal respiration, and rotenone plus antimycin A at 90 min to inhibit mitochondrial electron transport and determine non-mitochondrial respiration [33]. For ECAR measurement, glucose was injected at 30 min to initiate glycolysis, oligomycin at 60 min to stimulate maximal glycolytic capacity, and 2-DG at 90 min to inhibit glycolysis [33]. Key metabolic parameters were calculated from OCR and ECAR profiles. OCR parameters included basal respiration, ATP production, maximal respiration, spare respiratory capacity, proton leak, and non-mitochondrial respiration. ECAR parameters included basal glycolysis, glycolytic capacity, glycolytic reserve, and non-glycolytic acidification.

### 2.5. Statistical Analysis

Statistical analyses were performed using SPSS 27.0 (IBM, Armonk, NY, USA). The CCK-8 assay was performed with four independent replicates (*n* = 4), while all other experiments were performed with three independent replicates (*n* = 3). Differences among multiple groups were analyzed using one-way ANOVA followed by the least significant difference (LSD) post hoc test. Differences were considered statistically significant at *p* < 0.05.

## 3. Results

### 3.1. Effects of SVH and FSVH on HCT116 Cell Viability

**Figure 2:**
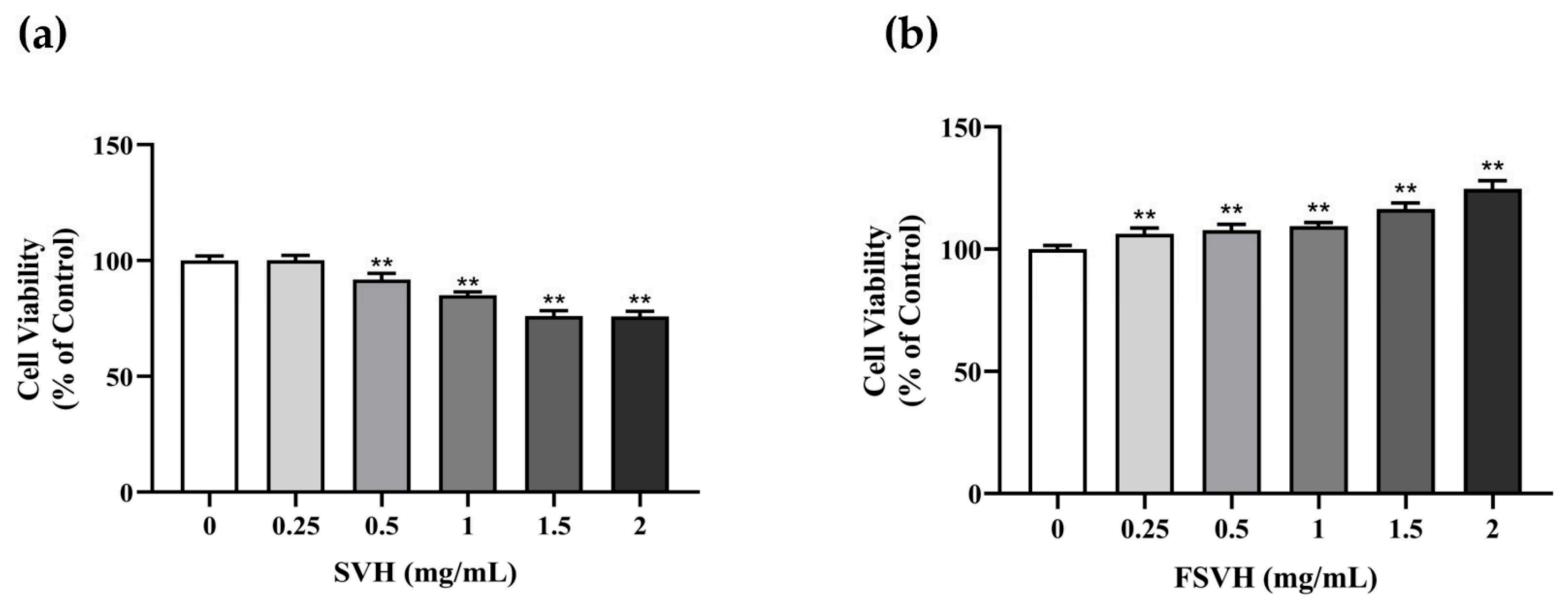
CCK-8 assay of viability in HCT116 cells treated with different concentrations of SVH and FSVH: (**a**) SVH treatment; (**b**) FSVH treatment. ** *p <* 0.01 vs. control.

Cell viability of HCT116 cells was evaluated using the CCK-8 assay after treatment with different concentrations of freeze-dried SVH and FSVH samples (0, 0.25, 0.5, 1, 1.5, and 2 mg/mL, based on dry weight) for 24 h. As shown in Figure 2, SVH and FSVH exhibited distinct effects on HCT116 cell viability. Compared with the control group, SVH treatment significantly reduced cell viability at concentrations of 0.5–2 mg/mL (*p* < 0.01), with similar cell viability values at 1.5 and 2 mg/mL (approximately 76%). In contrast, FSVH treatment significantly increased the CCK-8 signal at concentrations of 0.25–2 mg/mL compared with the control group (*p* < 0.01), although no significant differences were observed among different FSVH concentrations.

### 3.2. Effects of SVH and FSVH on Apoptosis in HCT116 Cells

The distribution of early and late apoptotic cells in HCT116 cells was analyzed by flow cytometry using Annexin V-FITC/PI staining after treatment with different concentrations of SVH and FSVH for 24 h. Cells were classified as Q2-1 (necrotic cells/cellular debris), Q2-2 (late apoptotic cells), Q2-3 (viable cells), and Q2-4 (early apoptotic cells). The total apoptosis rate was calculated as the sum of early and late apoptotic cells.

**Figure 3:**
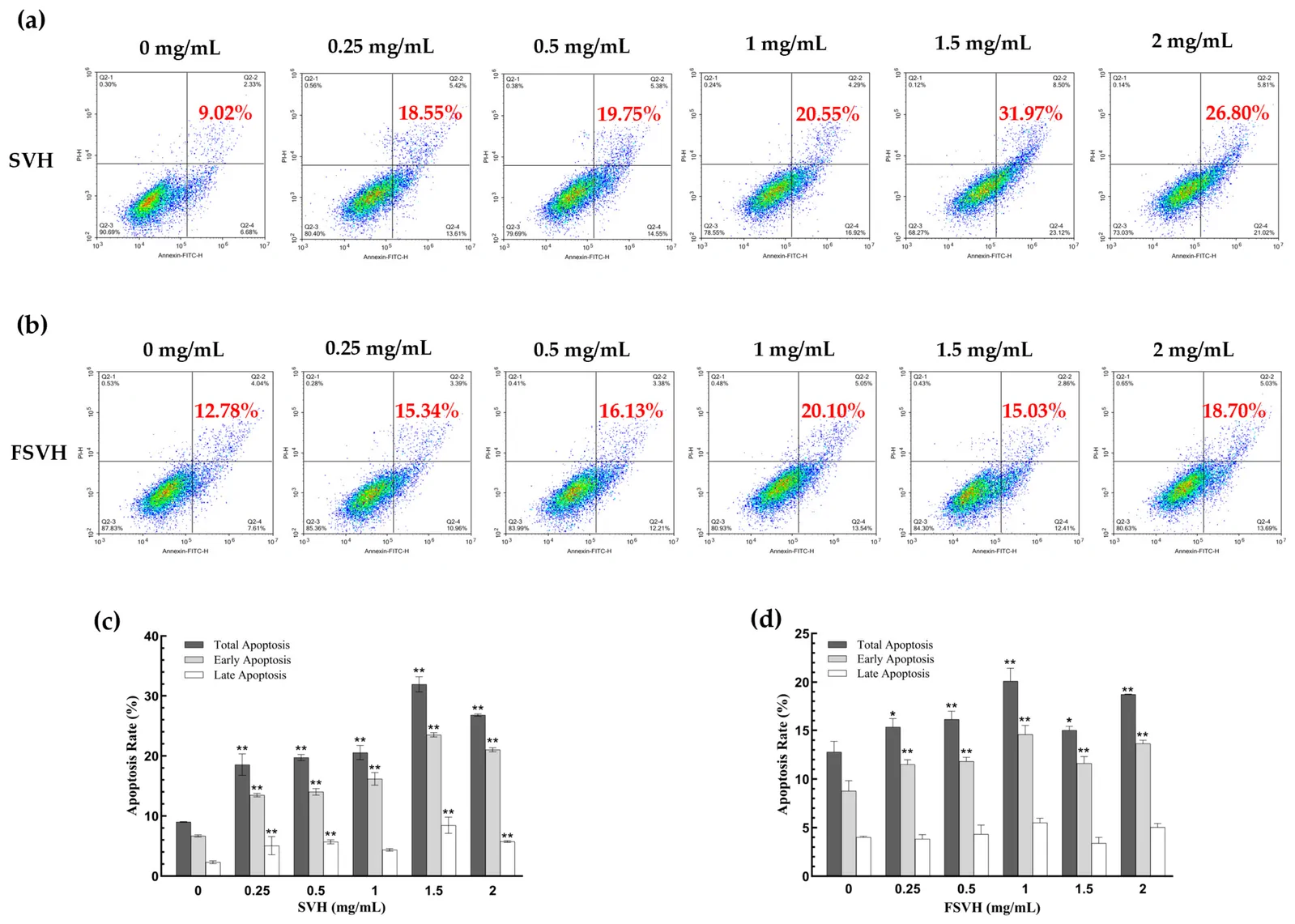
Flow cytometric analysis of apoptosis in HCT116 cells treated with different concentrations of SVH and FSVH: (**a**,**b**) representative flow cytometry plots; (**c**,**d**) quantitative analysis of apoptosis rates. Values in red represent the mean total apoptosis rates calculated from three independent experiments. * *p* < 0.05 vs. control and ** *p* < 0.01 vs. control.

SVH treatment induced apoptosis in HCT116 cells, as evidenced by the increased proportions of both early and late apoptotic cells detected by Annexin V-FITC/PI staining, with the highest total apoptosis rate observed at 1.5 mg/mL (Figure 3a,c). At this concentration, the total apoptosis rate increased from 9.02% in the control group to 31.97% (*p* < 0.01), accompanied by increases in early apoptotic cells from 6.69% to 23.50% (*p* < 0.01) and late apoptotic cells from 2.33% to 8.46% (*p* < 0.01). A slight decrease in total apoptosis rate was observed at 2 mg/mL SVH, although it remained significantly higher (*p* < 0.01) than that in the control group.

FSVH treatment also induced apoptosis in HCT116 cells, with the highest total apoptosis rate observed at 1 mg/mL (Figure 3b,d). At this concentration, the total apoptosis rate increased from 12.78% in the control group to 20.10% (*p* < 0.01), with early apoptotic cells increasing from 8.77% to 14.59% (*p* < 0.01) and late apoptotic cells increasing from 4.01% to 5.50%. Higher concentrations of FSVH did not further enhance apoptosis, although the total apoptosis rates at 1.5 and 2 mg/mL remained significantly higher than those in the control group (*p* < 0.05 and *p* < 0.01, respectively).

Overall, after 24 h treatment, SVH and FSVH treatments increased apoptosis in HCT116 cells under the tested conditions. Considering the combined results of cell viability and apoptosis assays, SVH (1.5 mg/mL) and FSVH (1 mg/mL), which induced clear apoptotic responses without excessive loss of cell viability, were selected for subsequent cellular function and energy metabolism analyses.

### 3.3. Effects of SVH and FSVH on Migration and Invasion of HCT116 Cells

**Figure 4:**
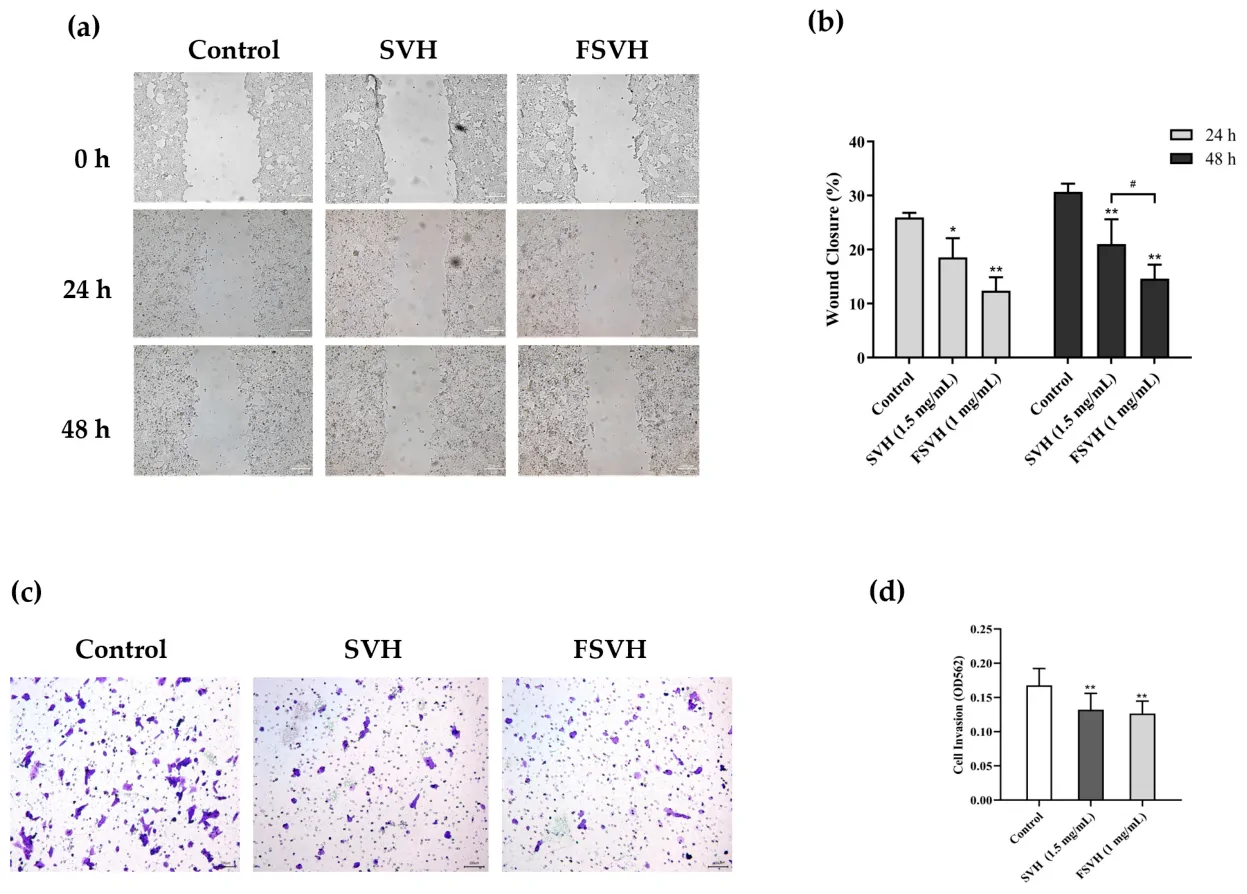
Wound healing and Transwell assays evaluating the migration and invasion of HCT116 cells treated with SVH (1.5 mg/mL) and FSVH (1 mg/mL): (**a**,**b**) representative images of the wound healing assay and corresponding wound closure rates at 0, 24, and 48 h; (**c**,**d**) representative images and quantitative analysis of cell invasion determined by the Transwell assay (absorbance at 562 nm). * *p* < 0.05 and ** *p* < 0.01 vs. control; ^#^ *p* < 0.05 SVH vs. FSVH.

Cell migration was assessed using a wound healing assay. As shown in Figure 4a,b, the control group showed the highest wound closure rate. Compared with the control group, both SVH and FSVH treatments significantly reduced the migratory capacity of HCT116 cells. After 24 h, wound closure was reduced by 28.44% and 52.27% following SVH and FSVH treatments, respectively, compared with the control group (*p* < 0.05 and *p* < 0.01, respectively). After 48 h, wound closure was reduced by 31.51% and 52.31% following SVH and FSVH treatments, respectively (both *p* < 0.01 vs. control), and FSVH treatment produced a significantly greater reduction in wound closure than SVH treatment (*p* < 0.05).

Cell invasion was assessed using a Transwell assay. As shown in Figure 4c,d, after 24 h treatment, both SVH and FSVH treatments significantly reduced the invasion ability of HCT116 cells compared with the control group (*p* < 0.01), with reductions of 21.21% and 24.52%, respectively. Overall, both SVH and FSVH inhibited the migration of HCT116 cells after 24 and 48 h treatment and reduced cell invasion after 24 h treatment under the tested conditions.

### 3.4. Effects of SVH and FSVH on Glucose Uptake and Lactate Production in HCT116 Cells

**Figure 5:**
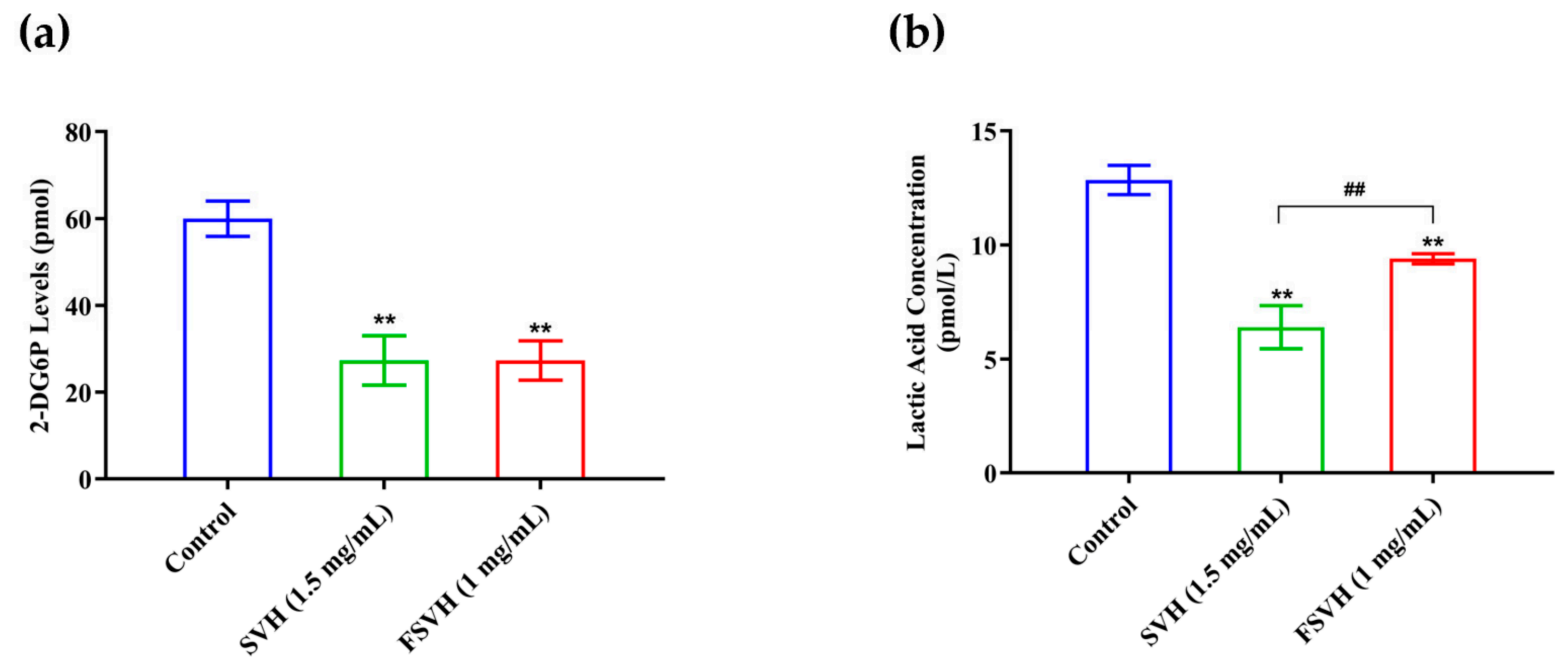
Glucose uptake and lactate production in HCT116 cells treated with SVH (1.5 mg/mL) and FSVH (1 mg/mL): (**a**) 2-DG6P levels; (**b**) lactic acid concentration. ** *p* < 0.01 vs. control; ^##^ *p* < 0.01 SVH vs. FSVH.

The effects of SVH and FSVH on glycolytic metabolism were evaluated in HCT116 cells by measuring glucose uptake and lactate production. As shown in Figure 5, both SVH (1.5 mg/mL) and FSVH (1 mg/mL) significantly reduced glucose uptake and lactate production compared with the control group (*p* < 0.01). Glucose uptake decreased by 54.41% and 54.40% following SVH and FSVH treatments, respectively (Figure 5a). Lactate production decreased by 50.21% and 26.86% following SVH and FSVH treatments, respectively (Figure 5b). Moreover, SVH treatment resulted in a significantly greater reduction in lactate production than FSVH treatment (*p* < 0.01). Overall, these findings indicated that both SVH and FSVH inhibited glucose uptake and lactate production in HCT116 cells.

### 3.5. Effects of SVH and FSVH on Mitochondrial Respiration in HCT116 Cells

**Figure 6:**
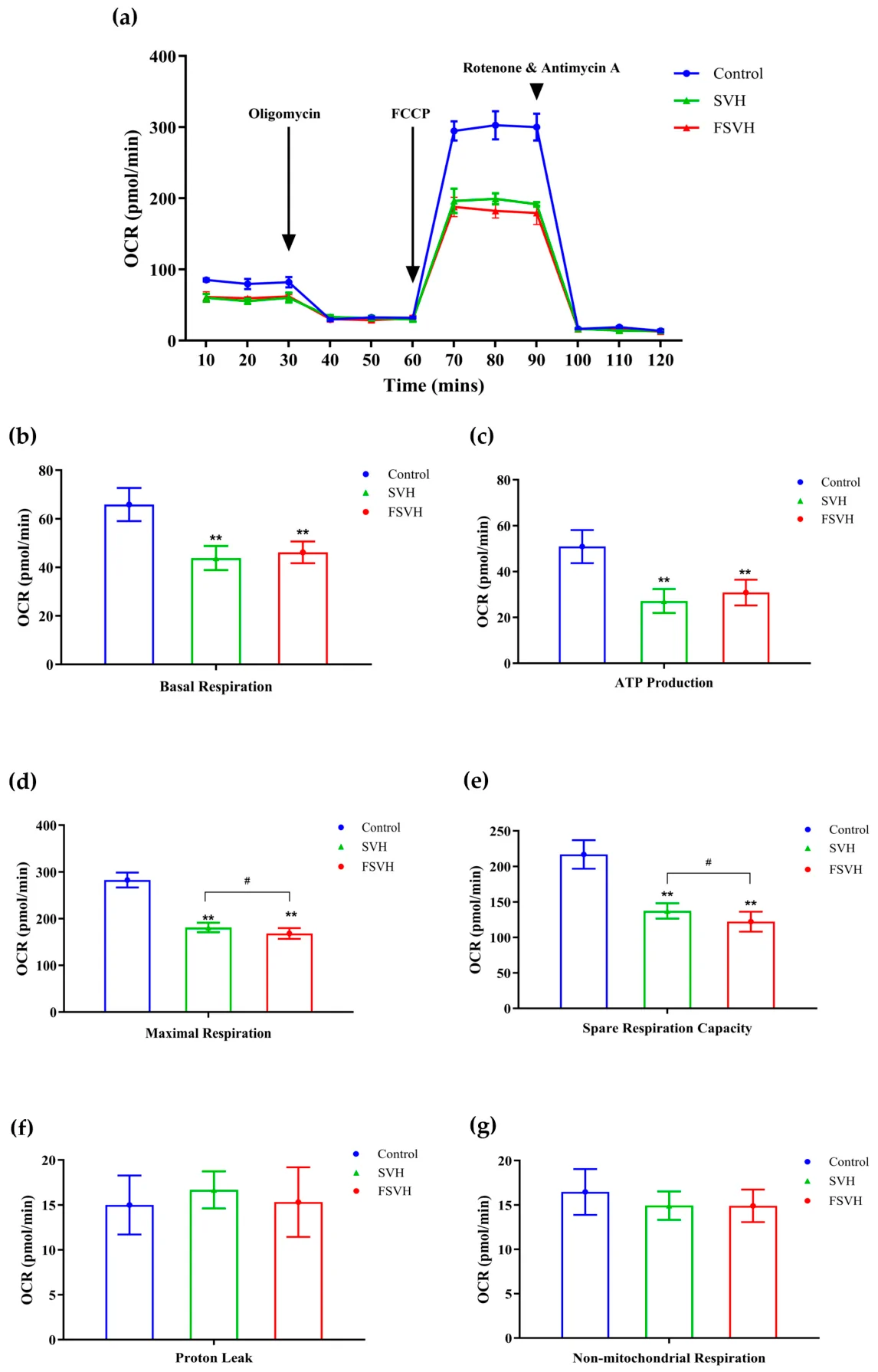
Mitochondrial respiration in HCT116 cells treated with SVH (1.5 mg/mL) and FSVH (1 mg/mL): (**a**) overall OCR profile; (**b**) basal respiration; (**c**) ATP production; (**d**) maximal respiration; (**e**) spare respiratory capacity; (**f**) proton leak; (**g**) non-mitochondrial respiration. ** *p* < 0.01 vs. control; ^#^ *p* < 0.05 SVH vs. FSVH.

Mitochondrial oxidative respiration plays an important role in ATP production and cellular energy metabolism in tumor cells. In this study, mitochondrial respiration was evaluated by measuring OCR using the mitochondrial stress test. As shown in Figure 6a, both SVH (1.5 mg/mL) and FSVH (1 mg/mL) treatments reduced the overall OCR profile of HCT116 cells, with more pronounced differences observed after FCCP injection.

Both SVH and FSVH treatments significantly reduced basal respiration and ATP production compared with the control group (*p* < 0.01). Basal respiration significantly decreased by 33.49% and 29.94% following SVH and FSVH treatments, respectively (Figure 6b); and ATP production decreased by 46.65% and 39.40%, respectively (Figure 6c). In addition, parameters associated with mitochondrial respiratory capacity were significantly decreased (*p* < 0.01). Maximal respiration decreased by 35.97% in the SVH group and 40.47% in the FSVH group compared with the control group (Figure 6d); and spare respiratory capacity decreased by 36.73% and 43.67%, respectively (Figure 6e). Especially, FSVH treatment resulted in significantly greater reductions in both maximal respiration and spare respiratory capacity than SVH treatment (*p* < 0.05). No significant changes were observed in proton leak (Figure 6f) or non-mitochondrial respiration (Figure 6g).

Overall, these results indicated that both SVH and FSVH impaired mitochondrial respiratory function in HCT116 cells, particularly by reducing respiratory capacity-related parameters. FSVH showed stronger effects than SVH on maximal respiration and spare respiratory capacity under the tested conditions.

### 3.6. Effects of SVH and FSVH on Glycolysis in HCT116 Cells

**Figure 7:**
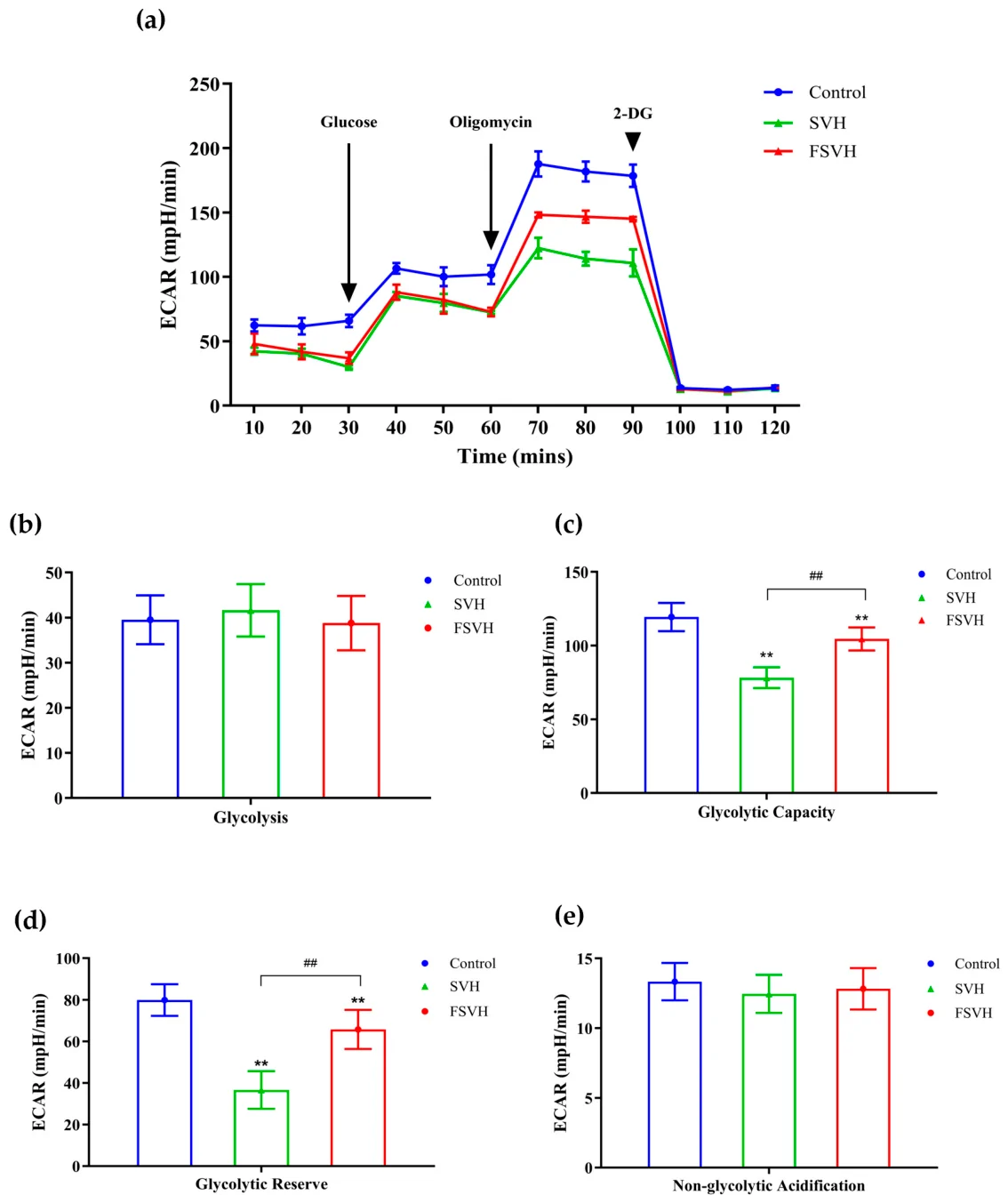
ECAR analysis of glycolytic activity in HCT116 cells treated with SVH (1.5 mg/mL) and FSVH (1 mg/mL): (**a**) overall ECAR profile; (**b**) basal glycolysis; (**c**) glycolytic capacity; (**d**) glycolytic reserve; (**e**) non-glycolytic acidification. ** *p* < 0.01 vs. control; ^##^ *p* < 0.01 SVH vs. FSVH.

To further evaluate the effects of SVH and FSVH on cellular glycolytic activity, the ECAR was measured in HCT116 cells. As shown in Figure 7a, treatment with SVH (1.5 mg/mL) or FSVH (1 mg/mL) reduced the overall ECAR profile compared with the control group.

Both treatments significantly reduced glycolytic capacity and glycolytic reserve (*p* < 0.01). Compared with the control group, glycolytic capacity decreased by 34.49% and 12.44% following SVH and FSVH treatments, respectively (Figure 7c); and glycolytic reserve decreased by 54.18% and 17.65%, respectively (Figure 7d). SVH treatment showed significantly greater reductions in both parameters than FSVH treatment (*p* < 0.01). Additionally, no significant changes were observed in basal glycolysis or non-glycolytic acidification after treatment (Figure 7b,e).

## 4. Discussion

Microbial fermentation may modify the composition and biological properties of food-derived substrates through microbial metabolism [27,34]. As reported in our previous study [28], *C. militaris* fermentation increased polysaccharide levels and resulted in the detection of cordycepin in FSVH (Appendix A), indicating changes in the bioactive composition of the hydrolysates. Previous studies have shown that fermentation can modify the functional properties of sea cucumber products, such as improving the antioxidant activity of peptides [20] and the biological properties of polysaccharides [35]. Similar findings have been reported for other *C. militaris*-fermented substrates, suggesting that fermentation may influence bioactive properties [27,36]. Therefore, the differences in bioactive composition between SVH and FSVH may contribute to their distinct cellular responses.

An experimental finding in this study was the increased CCK-8 signal following FSVH treatment. It should be noted that tetrazolium-based assays such as CCK-8 primarily reflect cellular metabolic activity and reducing capacity rather than directly representing the number of viable cells [37,38]. Therefore, the increased CCK-8 signal after FSVH treatment may reflect alterations in cellular metabolic activity or reducing capacity rather than increased cell proliferation. Meanwhile, apoptosis analysis showed that FSVH treatment also increased apoptosis in HCT116 cells, suggesting that increased CCK-8 signal and apoptosis induction may occur simultaneously under the tested conditions. Thus, the elevated CCK-8 signal should not be interpreted as enhanced cell survival or proliferation. Further investigations may provide additional insights into this phenomenon.

Apoptosis is a programmed cell death process essential for maintaining cellular homeostasis and is frequently dysregulated in cancer cells due to alterations in mitochondrial function [39,40]. In this study, both SVH and FSVH significantly increased apoptosis in HCT116 cells, indicating that sea cucumber viscera hydrolysates exerted pro-apoptotic effects under the tested conditions. These effects may be associated with the bioactive components present in the hydrolysates. Previous studies have suggested that *C. militaris* polysaccharides may induce apoptosis through ROS accumulation and activation of endogenous apoptotic signaling pathways, including the upregulation of pro-apoptosis proteins Bax and caspase-3 as well as the downregulation of the anti-apoptosis protein Bcl-2 [41]. Other bioactive compounds reported from *C. militaris*, such as cordycepin, have also been reported to regulate apoptosis through Bax-dependent mitochondrial pathways and modulation of MYC expression and CD47 signaling [42,43,44]. Although these mechanisms were not directly examined in the present study, they may provide possible explanations for the apoptotic effects observed after SVH and FSVH treatment. Furthermore, both hydrolysates impaired mitochondrial respiration in HCT116 cells by reducing basal respiration, maximal respiration, ATP production, and spare respiratory capacity, which may be associated with increased cellular susceptibility to apoptosis.

Besides the effects on apoptosis, SVH and FSVH also exhibited inhibitory effects on the migration and invasion of HCT116 cells. Similar inhibitory effects have also been reported for sea cucumber extracts including polysaccharides and peptides, which can suppress adhesion, migration, and invasion in different cancer cell models [45,46,47]. Cell migration and invasion are energy-intensive processes that require continuous ATP production to support cytoskeletal remodeling and interactions with the extracellular matrix [48]. Previous studies have shown that inhibition of mitochondrial respiration or glycolysis can significantly impair migration and invasion in cancer cells, highlighting the importance of cellular metabolic regulation in tumor progression and aggressiveness [48]. In the present study, both SVH and FSVH suppressed the migration and invasion of HCT116 cells at concentrations that also altered cellular energy metabolism. These findings suggest that the reduced migratory and invasive potential of CRC cells may be associated with metabolic alterations induced by sea cucumber viscera hydrolysates.

Metabolic reprogramming is a hallmark of cancer cells, involving coordinated alterations in glycolysis and mitochondrial oxidative phosphorylation to maintain energy production and metabolic adaptation [30,31]. In this study, SVH and FSVH exhibited distinct effects on cellular energy metabolism, suggesting that fermentation altered the metabolic regulatory properties of the hydrolysates rather than simply enhanced or reduced their overall activity. SVH showed stronger inhibitory effects on glycolytic parameters, including lactate production, glycolytic capacity, and glycolytic reserve. As an enzymatic hydrolysate, SVH contains peptides and other soluble components released during enzymatic hydrolysis, which may contribute to its metabolic regulatory effects. During fermentation, microbial metabolism may transform existing components or introduce new bioactive constituents, thereby altering the contribution of different components to metabolic regulation and leading to altered metabolic effects in FSVH. Therefore, the weaker glycolytic inhibition by FSVH may reflect a shift in metabolic regulation rather than reduced activity, with FSVH showing greater effects on mitochondrial respiratory function.

Reduced mitochondrial respiratory capacity and spare respiratory capacity reflect impaired mitochondrial function and a decreased ability of cells to adapt to increased energy demands or metabolic stress [49]. In the present study, FSVH showed greater inhibitory effects on mitochondrial respiratory parameters, which may reduce the metabolic flexibility of HCT116 cells and limit their ability to maintain energy homeostasis under stress conditions. The distinct metabolic regulatory profiles of SVH and FSVH suggest that fermentation could be used to modulate the functional characteristics of hydrolysates, providing a basis for their targeted development according to specific application needs. Further studies may help clarify the bioactive components and mechanisms underlying these differential metabolic effects.

This cell-based study evaluated the application potential of sea cucumber viscera hydrolysates after *C. militaris* fermentation by analyzing their effects on cellular functions and energy metabolism, suggesting that fermentation may modify their functional properties and provide new possibilities for the application of sea cucumber processing by-products. However, several aspects of this study could be further explored to better understand their biological effects and application potential. First, this study was limited to the HCT116 cell line, and validation in additional cell models may provide further insights into the selectivity and safety of SVH and FSVH. Second, the mechanisms underlying the differential cellular responses induced by SVH and FSVH remain to be further elucidated. Third, SVH and FSVH were compared at their selected effective concentrations rather than at identical concentrations, which should be considered when interpreting their differences. Finally, these findings were obtained from an in vitro model and require further validation in more complex biological systems.

## 5. Conclusions

In summary, both hydrolysates induced apoptosis and inhibited the migration and invasion of HCT116 cells, accompanied by alterations in mitochondrial respiration and glycolytic metabolism. Although SVH and FSVH exhibited comparable effects on several cellular functions, their effects on energy metabolism differed under the selected experimental conditions. SVH showed stronger effects on glycolysis-related parameters, whereas FSVH showed greater effects on mitochondrial respiratory capacity. These findings suggest that *C. militaris* fermentation may have modified the biological properties of sea cucumber viscera hydrolysates, which may contribute to the differential cellular responses induced by SVH and FSVH. This study provides insights into the potential functional applications of sea cucumber viscera hydrolysates and supports further exploration of their active components and underlying mechanisms.

## Data Availability

The original contributions presented in this study are included in the Supplementary Material. Further inquiries can be directed to the corresponding authors.

## Supplementary Materials

The following supporting information can be downloaded at: https://www.mdpi.com/article/10.3390/foods15173065/s1. Table S1: Raw data of cellular function and energy metabolism assays in HCT116 cells treated with FSVH and SVH.

## Abbreviations

The following abbreviations are used in this manuscript:

2-DG	2-deoxyglucose
2-DG6P	2-deoxyglucose-6-phosphate
CCK-8	Cell Counting Kit-8
CRC	Colorectal cancer
DMEM	Dulbecco’s modified Eagle’s medium
ECAR	Extracellular acidification rate
FCCP	Carbonyl cyanide-4-(trifluoromethoxy)phenylhydrazone
FSVH	Fermented sea cucumber viscera hydrolysates
HPLC	High-performance liquid chromatography
OCR	Oxygen consumption rate
PBS	Phosphate-buffered saline
ROS	Reactive oxygen species
SVH	Sea cucumber viscera hydrolysates

## Appendix A. The Contents of FSVH and SVH

The major components of FSVH and SVH are shown in Table A1, as reported in our previous study [28]. All contents were determined based on the dry weight of the freeze-dried SVH and FSVH samples. Compared with SVH, the protein and peptide content of FSVH decreased to 32.35 g/100 g, while the polysaccharide content significantly increased to 19.53 g/100 g (*p* < 0.05). There was no significant difference in saponin content between SVH and FSVH (*p* > 0.05). Cordycepin content was 852.19 mg/kg in FSVH (*p* < 0.05), whereas it was not detected in SVH. These findings indicate that fermentation with *C. militaris* altered the bioactive composition of FSVH.

**Table A1:** Contents of bioactive compounds in SVH and FSVH (dry weight basis).

Bioactive Compounds	SVH	FSVH
Protein/peptide (g/100 g)	37.05 ± 2.89	32.35 ± 3.89
Polysaccharide (g/100 g)	13.85 ± 2.25	19.53 ± 2.56 *
Saponins (mg/kg)	5187.42 ± 339.15	5537.93 ± 323.14
Cordycepin (mg/kg)	0	852.19 ± 83.54 *

Note: Data are presented as mean ± SD (*n* = 3). * *p* < 0.05 SVH vs. FSVH.
